# Small Extracellular Vesicles from Head and Neck Squamous Cell Carcinoma Cells Carry a Proteomic Signature for Tumor Hypoxia

**DOI:** 10.3390/cancers13164176

**Published:** 2021-08-19

**Authors:** Alicja Głuszko, Mirosław J. Szczepański, Theresa L. Whiteside, Torsten E. Reichert, Jacek Siewiera, Nils Ludwig

**Affiliations:** 1 Chair and Department of Biochemistry, Medical University of Warsaw, 1 Banacha St., 02-097 Warsaw, Poland; alicja.gluszko@wum.edu.pl; 2 Department of Otolaryngology, Centre of Postgraduate Medical Education, 03-242 Warsaw, Poland; 3 Department of Pathology, University of Pittsburgh School of Medicine, Pittsburgh, PA 15213, USA; whitesidetl@upmc.edu; 4 Department of Immunology and Otolaryngology, University of Pittsburgh School of Medicine, Pittsburgh, PA 15213, USA; 5 Department of Pathology, UPMC Hillman Cancer Centre, Pittsburgh, PA 15213, USA; 6 Department of Oral and Maxillofacial Surgery, University Hospital Regensburg, 93053 Regensburg, Germany; torsten.reichert@ukr.de (T.E.R.); nils.ludwig@ukr.de (N.L.); 7 Department of Hyperbaric Medicine, Military Institute of Medicine, 04-141 Warsaw, Poland; jsiewiera@wim.mil.pl

**Keywords:** small extracellular vesicles, exosomes, hypoxia, proteomics, HNSCC

## Abstract

**Simple Summary:**

Tissue hypoxia is a hallmark of head and neck squamous cell carcinoma (HNSCC) and is considered to drive tumor progression and resistance to anti-cancer therapies. The aim of our study was to characterize the influence of hypoxic environments on the release and proteomic cargo composition of small extracellular vesicles (sEVs). We demonstrated in three HNSCC cell lines that sEV secretion is enhanced in response to hypoxic conditions and that hypoxic sEVs carry distinct proteomic profiles, which can not only discriminate between normoxic and hypoxic conditions, but also discriminate between various degrees of tissue hypoxia. Therefore, sEVs are a potential resource for monitoring tissue hypoxia in HNSCC or even anti-angiogenic or vessel normalization therapies.

**Abstract:**

Tissue hypoxia is commonly observed in head and neck squamous cell carcinomas (HNSCCs), resulting in molecular and functional alterations of the tumor cells. The aim of this study was to characterize tumor-derived small extracellular vesicles (sEVs) released under hypoxic vs. normoxic conditions and analyze their proteomic content. HNSCC cells (FaDu, PCI-30, SCC-25) and HaCaT keratinocytes were cultured in 21, 10, 5, and 1% O_2_. sEVs were isolated from supernatants using size exclusion chromatography (SEC) and characterized by nanoparticle tracking analysis, electron microscopy, immunoblotting, and high-resolution mass spectrometry. Isolated sEVs ranged in size from 125–135 nm and contained CD63 and CD9 but not Grp94. sEVs reflected the hypoxic profile of HNSCC parent cells: about 15% of the total detected proteins were unique for hypoxic cells. Hypoxic sEVs expressed a common signature of seven hypoxia-related proteins (KT33B, DYSF, STON2, MLX, LIPA3, NEK5, P12L1) and were enriched in pro-angiogenic proteins. Protein profiles of sEVs reflected the degree of tumor hypoxia and could serve as potential sEV-based biomarkers for hypoxic conditions. Adaptation of HNSCC cells to hypoxia is associated with increased release of sEVs, which are enriched in a unique protein profile. Thus, tumor-derived sEVs can potentially be useful for evaluating levels of hypoxia in HNSCC.

## 1. Introduction

Head and neck squamous cell carcinomas (HNSCCs) are clinically complex and molecularly heterogenous [1]. Despite intensive studies of HNSCC and the development of new therapeutic modalities, the five-year survival rate has remained at 50% for decades [2,3,4]. One hallmark of HNSCC is the presence of hypoxic areas within the tumor tissue. Further, levels of tissue hypoxia correlate with poor prognosis and reduced response to therapy in patients with HNSCC [5,6]. Hypoxia is commonly defined as oxygen tension pO_2_ ≤ 10 mmHg. However, this value might vary depending on the tumor stage and size as well as methods of measurement. Oxygenation levels inside the tumor usually range from 1 to 2% O_2_ and are lower compared to levels in non-malignant tissues [5,7]. It is well established that tumor cells adapt to low oxygen levels, often acquiring a more aggressive phenotype. During this adaptation, several intracellular signaling pathways are activated, leading to proteomic/genomic changes that ultimately alter their functional behavior [8,9]. The most frequently described oxygen-dependent regulatory component of the adaptation process is the hypoxia inducible factor (HIF), which is upregulated in most tumor tissues and is associated with increased expression of genes regulating cell growth, survival, and proliferation [10,11,12].

Hypoxic conditions stimulate release of small extracellular vesicles (sEVs) from tumor cells [13,14]. sEVs are a subset of EVs sized at 30 to 150 nm that are derived from the endocytic compartment of parent cells via the fusion of multivesicular bodies (MVB) with the plasma membrane. They are abundantly produced by tumor cells and are enriched in the plasma of HNSCC patients [15]. The molecular content and functions of sEVs in plasma of HNSCC patients correlate with disease activity [16]. These sEVs mediate immunosuppressive as well as pro-angiogenic functions in the tumor microenvironment (TME) and at distant sites [16,17]. sEVs carry a complex cargo consisting of proteins, lipids, glycans, and various nucleic acids. A recent large-scale comparative analysis of sEVs from individuals with or without cancer has identified the tumor-associated protein profiles that discriminated cancer from non-cancer [18]. Further, analyses in human cancers of plasma-derived sEVs and paired tumor tissue-derived sEVs confirmed their proteomic similarity, validating the role of sEVs as a new, non-invasive liquid biopsy in cancer [18,19].

Hypoxic conditions within the tumor appear to have a major impact on the molecular content and biological functions of tumor cell-derived sEVs. Importantly, pro-angiogenic factors such as matrix metalloproteinases (MMPs), platelet-derived growth factor (PDGF), and caveolin-1 were found to be enriched in sEVs produced in hypoxic conditions. The enrichment in these proteins in hypoxic sEVs resulted in reprogramming of endothelial cells and an increased production of growth factors and cytokines, ultimately stimulating tumor angiogenesis [20,21,22]. Moreover, several immunosuppressive proteins including PD-1, PD-L1, and CTLA-4 were found to be enriched in hypoxic sEVs, contributing to hypoxia-dependent modulation of anti-tumor immune responses [23,24]. These and other studies suggest that hypoxia induces major changes in the release of sEVs by the tumor and in their contents [21,25,26].

The aim of this study was to analyze the proteomes of sEVs derived from HNSCC cell lines and keratinocytes cultured in normoxic or hypoxic conditions and evaluate oxygenation-related changes in the sEV cargo composition. We asked whether (1) the sEV proteome reflects the oxygenation status of the parent cells; (2) hypoxia-derived sEVs carry unique proteomic signatures, which could be used as potential biomarkers of tissue hypoxia; (3) sEVs reflect the degree of hypoxia in tumor cells; and (4) the proteome of sEVs produced and released under hypoxic conditions is enriched in pro-angiogenic factors.

## 2. Materials and Methods

### 2.1. Cell Lines and Cell Culture

FaDu, SCC-25, and HaCat cell lines were purchased from American Cell Type Collection (ATCC) and the PCI-30 cell line was obtained from Dr. Theresa L. Whiteside (UPMC Hillman Cancer Center, Pittsburgh, PA, USA). Detailed information with regards to HNSCC cell lines is provided in Table S1. Cells were grown in RPMI 1640 (Gibco) supplemented with 1% (*v*/*v*; concentration of 10 mL/L according to manufacturer’s recommendations) penicillin/streptomycin and 10% (*v*/*v*) exosome-depleted FBS (Gibco) at 37 °C and in the atmosphere of 5% CO_2_ in air. For experiments in hypoxia, cells were exposed to a humidified atmosphere of 1, 5, or 10% O_2_ and 5% CO_2_ at 37 °C in a BioSpherix Xvivo System Model X3.

### 2.2. Isolation of sEVs

For sEV isolation, 2.5 × 10^6^ cells were seeded under normoxic or hypoxic conditions with 25 mL media in 150 cm^2^ cell culture flasks as previously described [27]. Supernatants were collected by decanting after 72 h. Pre-clearing of supernatants was performed by centrifugation at room temperature (RT) for 10 min at 2000× *g* (removal of cell debris and larger apoptotic bodies) and subsequently at 4 °C for 30 min at 10,000× *g* (removal of large microvesicles and smaller apoptotic bodies), followed by filtration using a 0.22 µm bacterial filter (removal of EVs with diameters of 200–500 nm). Filtered supernatants were concentrated at 2000× *g* and 1 mL of the concentrate was placed on a size exclusion chromatography (SEC) column with Sepharose CL-2B (GE Healthcare Bio-Sciences, Marlborough, MA, USA). sEVs were eluted in 1 mL fractions using PBS, and fraction #4 was harvested for downstream applications as previously described [28].

### 2.3. Cryogenic Electron Microscopy (Cryo-EM)

Direct visualization of sEVs was performed using Cryo-EM. sEVs were concentrated using 100 K Amicon Ultra 2 mL concentrators (Merck) at 4000× *g* for 30 min at RT. A total of 3 µL of concentrated sEVs were applied on lacey carbon EM grids, which were previously glow-discharged (30 s, 25 mA) in a Pelco EasiGlow system, and blotted for 2 s, followed by plunge-freezing into precooled liquid ethane with Vitrobot (Thermo Fisher, Waltham, MA, USA). Obtained samples, embedded in a thin layer of amorphous ice, were preserved from radiation damage and studied in native state in a 200 kV Glacios cryo-electron microscope (Thermo Fisher), equipped with a high sensitive direct electron detector (DED) Falcon 3EC (Thermo Fisher) at accelerating voltage of 200 kV. Images were obtained at 72,000× magnification in linear mode with the defocus value in the range of [−2 µm; −5 µm]. The accumulated total dose per image did not exceed 50 e^−^/A^2^ and to minimize radiation damage during image acquisition low-dose mode was used. The EPU 2.7 software (Thermo Fisher) was used for single particle analysis.

### 2.4. Nanoparticle Tracking Analysis (NTA)

Determinations of size and quantifications of sEVs were performed using ZetaView, equipped with the NTA analytical software (version 2.3, Particle Metrix GmbH, Inning am Ammersee, Germany). For each sample, three biological replicates were analyzed.

### 2.5. Western Blotting

Protein concentrations of sEV samples were measured using a BCA protein assay (Pierce Biotechnology, Waltham, MA, USA). Proteins were separated by 12% SDS-PAGE in reducing or non-reducing conditions and 10 ug protein aliquots/lane were transferred onto a PVDF 0.2 µm membrane (Millipore, Burlington, MA, USA) followed by blocking with 5% non-fat milk. Incubation with primary antibodies anti-CD63 (1:400, Invitrogen, Waltham, MA, USA, 10628D), anti-CD9 (1:1000, Invitrogen, 10626D), and anti-Grp94 (1:1000, Thermo Fisher, 36-2600) was performed overnight at 4 °C, followed by incubation with secondary HRP-conjugated antibody (1:1000 in 5% non-fat milk, anti-rabbit, anti-mouse, Cell Signalling Technology, Danvers, MA, USA) for 1 h at RT. Visualization was performed by chemiluminescence ChemiDoc.

### 2.6. Sample Preparation for Mass Spectrometry (MS)

A total of 10 ug of proteins from each sample (sEV fractions and cell lysates) were precipitated using ice cold (−20 °C) Acetonitrile (ACN, Merck, Kenilworth, NJ, USA) in a 1:4 ratio and centrifuged at −9 °C for 30 min at 18,000× *g*. Supernatants were removed and excess of ACN was evaporated using a vacuum centrifuge (5 min, RT). The protein pellet was dissolved in 40 mM ammonium bicarbonate. Reduction and alkylation were carried out using 500 mM DTT (in a final concentration of 20 mM) and 1 M IAA (in a final concentration of 40 mM). Proteins were incubated at 37 °C for 16 h in-solution with Trypsin Gold (Promega, Madison, WI, USA) for digestion. Digested samples were diluted with 0.1% formic acid (Thermo Fisher) and centrifuged at 2 °C for 30 min at 18,000× *g* followed by loading of 150 ng of protein to nanoUHPLC separation. For all samples, three biological replicates were analyzed.

### 2.7. Protein Identification and Quantitation by MS

LC-MS analysis was carried out using the nanoUHPLC (nanoElute, Bruker, Billerica, MA, USA) coupled by CaptiveSpray (Bruker) to ESI-Q-TOF mass spectrometer (Compact, Bruker). Two-Column separation method was used, i.e., pre-column (300 µm × 5 mm, C18 PepMap 100, 5 µm, 100 Å, Thermo Scientific) and Aurora separation column with CSI fitting (75 µm × 250 mm, C18 1.6 µm) in a gradient 2% B to 35% B for 90 min using 300 nL/min flow rate. Following mobile phases were used: A—0.1% formic acid in water; B—0.1% formic acid in ACN.

Ionization of the samples was carried out at a gas flow of 3.0 L/min, temperature of 150 °C and voltage of the capillary 1600 V. The quadrupole energy was set to 5.0 eV and collision chamber energy 7.0 eV with an ion transfer time of 90 µs. The ions were analyzed in the positive polarity mode in the range 150–2200 *m*/*z*, with the acquisition frequency of the 1 Hz spectrum, as well as with the auto MS/MS system.

The collected spectra were analyzed and calibrated (Na Formate) in DataAnalysis software (Bruker) and after extraction of the peak list, identified in ProteinScape (Bruker) using the MASCOT server. Relative intensity was measured using the ProteinScape 4.0 software (Bruker) based on chromatographic pick size and MS/MS spectrum intensity of each identified polypeptide. Proteins were identified using the online SwissProt and NCBI_prot databases. Generated mass spectrometry raw data are deposited in the publicly available repository Proteomics Identification Database at the link: https://wum-my.sharepoint.com/:f:/g/personal/alicja_gluszko_wum_onmicrosoft_com/EhMdBsd5-TpLpTWwKAWrED8BKDnNEnCpaLcimBwX_IyUvw?e=munWZb, accessed on 13 August 2021 with following accession: data set identifier PID13082021, password 13082021.

Bioinformatics analysis were performed to predict protein profile annotations using FunRich (http://www.funrich.org/, accessed on 30 March 2021), Gene Ontology (http://geneontology.org, accessed on 30 March 2021), Uniprot (https://www.uniprot.org/, accessed on 30 March 2021), KEGG (https://www.genome.jp/kegg/, accessed on 30 March 2021), STRING (https://string-db.org/, accessed on 30 March 2021) databases, and PANTHER Classification System (http://www.pantherdb.org/, accessed on 30 March 2021). Analysis was performed using the Functional Enrichment analysis tool (version 3.1.4, a standalone tool [29]. *p* < 0.05 was considered to indicate a statistically significant difference.

### 2.8. Wound Healing Assay

Subconfluent HUVEC monolayers in 48-well plates were starved for 24 h followed by treatment with 3 µg of sEVs per well and incubation for 24 h. The resulting confluent monolayers were mechanically wounded with pipet tips and captured with an inverted microscope (Zeiss Observer Z1, Axiovision 4.8 software; illumination system LUMEN 200; PRIOR, Germany) at 5× magnification. Capturing was repeated at 16 h after the initial scratch. Experiments were performed in triplicates and results calculated as a percentage of recovery using ImageJ.

### 2.9. Statistical Analysis

Values are expressed as mean ± standard deviation (SD). Differences between groups were assessed by Student *t* test, ANOVA or Kruskal-Wallis one-way analysis of variance. To isolate differences between groups, adequate post hoc tests were performed. Differences were considered significant at *p* < 0.05.

## 3. Results

### 3.1. Characterization of sEVs

**Figure 1:**
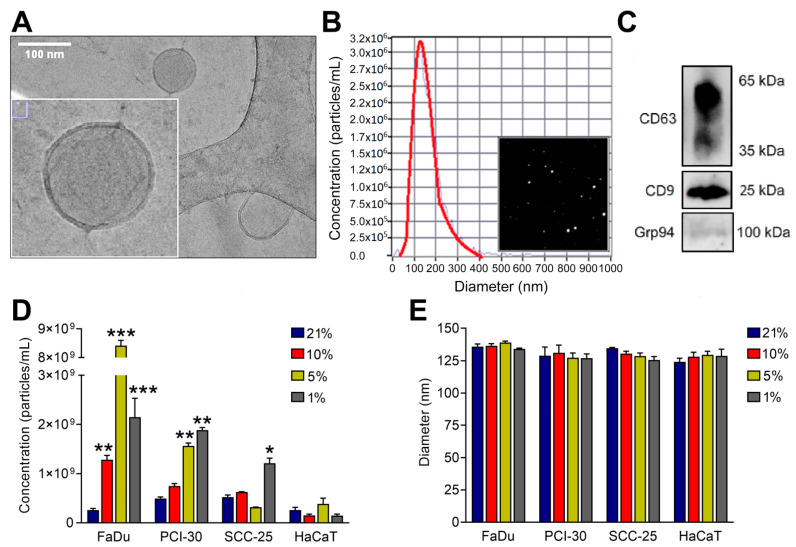
Characterization of small extracellular vesicles (sEVs) derived from human Head and Neck Squamous Cell Carcinoma cell lines: FaDu (hypopharyngeal squamous cell carcinoma), PCI-30 (tongue squamous cell carcinoma), SCC-25 (tongue squamous cell carcinoma); and sEVs derived from HaCat (immortalized human keratinocytes). (**A**) Representative cryogenic transmission microscopy image of HNSCC cell-derived sEVs. (**B**) Representative concentration and size distribution plot of HNSCC-derived sEVs measured by nanoparticle tracking analysis (NTA) and particle visualization based on Brownian motions. (**C**) Immunoblotting of sEV markers CD63, CD9, and negative marker Grp94 in HNSCC-derived sEVs. Full blot images are presented in Figures S2–S4. (**D**) Particle concentration related to normoxic (21% O_2_) and hypoxic (1% O_2_, 5% O_2_, 10% O_2_) conditions. Results were obtained using NTA and normalized to the protein levels in lysates of producer cells. (**E**) Particle diameter related to normoxic (21% O_2_) and hypoxic (1% O_2_, 5% O_2_, 10% O_2_) conditions. Results were obtained using NTA. All data represent three biological replicates and are presented as means ± SD. * *p* < 0.05 vs. 21%; ** *p* < 0.01 vs. 21%; *** *p* < 0.001 vs. 21%.

sEVs were isolated from supernatants of HNSCC cell lines FaDu, PCI-30, and SCC-25 (cell lines are characterized in Table S1) as well as the keratinocyte cell line HaCaT using SEC and characterized in accordance with the MISEV2018 guidelines [30]. Purified sEVs visualized by Cryo-EM showed the typical vesicular morphology with mean diameters of 30–150 nm and 4 nm lipid bilayer membranes (Figure 1A). By NTA, mean particle diameters varied from 125–135 nm (Figure 1B) and immunoblotting analysis demonstrated the presence of sEV markers CD63 and CD9, as well as the absence of the negative sEV marker, Grp94 (Figure 1C). The concentration of isolated sEVs derived from cells exposed to normoxic (21% O_2_) and hypoxic (10%, 5% or 1% O_2_) conditions by NTA was significantly increased in FaDu cells cultured in hypoxic conditions, with highly elevated levels of sEV concentrations for cells cultured in 5% O_2_. For PCI-30 cells, a dose-dependent increase of sEV concentrations was observed with significantly elevated numbers for cells cultured in 5 and 1% O_2_. SCC-25 cells showed no alterations of sEV concentrations when cultured in 10 and 5% O_2_, but had significantly elevated sEV concentration when being cultured in 1% O_2_ (Figure 1D). HaCaT cells, which were used as non-malignant cell control, showed no alterations of sEV concentrations in response to hypoxia (Figure 1D), indicating that the release of sEVs by HNSCC cells is more rigorously regulated by oxygen levels compared to their non-malignant counterparts. Although the numbers of released sEVs was elevated in the HNSCC cell lines, the sEV characteristics remained identical regardless of the oxygenation status of the cells (Figure 1E and Figure S1). The vesicle diameter measured by NTA was not altered by hypoxic culture conditions.

### 3.2. sEVs Reflect the Hypoxic State of HNSCC Cells and Carry Unique Hypoxia-Related Protein Signatures

**Figure 2:**
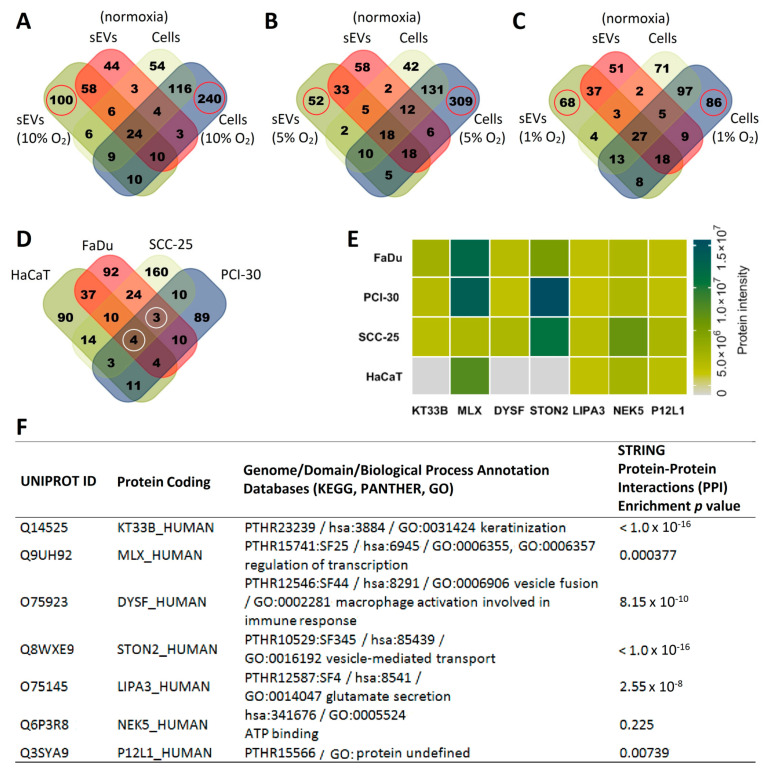
The protein profile of small extracellular vesicles (sEVs) reflects the hypoxic state of Head and Neck Squamous Cell Carcinoma (HNSCC) cells and can be utilized to identify unique hypoxia-related protein signatures in sEVs. The numbers of proteins identified in sEVs reflect the hypoxic state of HNSCC cells. Venn diagrams show numbers of proteins detected in cells cultured in 21% O_2_ (normoxia) in comparison to cells and sEVs obtained from supernatants of the cells exposed to (**A**) 10% O_2,_ (**B**) 5% O_2_, and (**C**) 1% O_2_. (**A**–**C**) Unique proteins are highlighted by red circles in the Venn diagrams presenting SCC-25 as a representative cell line. (**D**) Proteins detected in sEVs derived from HNSCC cell lines FaDu, PCI-30, and SCC-25 exposed to 10, 5, and 1% O_2_, but absent in 21% were grouped, and shared proteins are presented at the Venn diagram. Seven common proteins were identified (white circles). (**E**) Mean abundance of the 7 common proteins encoded with genes: KT33B (Keratin 33B, Type I), MLX (Max-like protein X), DYSF (Dysferlin), STON2 (Stonin-2), LIPA3 (Liprin-alpha-3), NEK5 (Serine/threonine-protein kinase Nek5), P12L1 (Putative POM121-like protein 1) detected only in hypoxia-derived HNSCC sEVs and Human Keratinocyte (HaCaT)-derived sEVs. (**F**) Information with regards to protein identification number, genome, domain, biological process annotations and protein–protein interactions is based on Universal Protein Resource (UNIPROT), Kyoto Encyclopedia of Genes and Genomes (KEGG), Protein Analysis Through Evolutionary Relationships (PANTHER), Search Tool for the Retrieval of Interacting Genes/Proteins (STRING), and Gene Ontology (GO) classification systems.

To investigate the proteomic cargo components of sEVs and compare them to the proteome of their parent cells, MS analysis was performed. The proteomic profile of HNSCC cells and keratinocytes and of sEVs isolated from these cells was altered, depending on the oxygenation status of the cells. Hypoxic cells or sEVs contained a variety of unique proteins that were not shared with normoxic cells or sEVs. Unique proteins are highlighted by red circles in the Venn diagrams in Figure 2A–C presenting the SCC-25 cell line as a representative. These results show that the hypoxia-triggered proteomic changes in cells are at least partly recapitulated in the protein composition of sEVs. To define a protein signature, which is uniquely present in sEVs derived from hypoxic cultures and, therefore, potentially discriminates normoxic from hypoxic sEVs, HNSCC cell line-derived sEV proteins were analyzed and only the set of proteins which were present in hypoxia-derived sEVs, but not in normoxia-derived sEVs, were included in the further analysis. As highlighted by white circles in Figure 2D, the sEVs derived from FaDu, PCI-30, and SCC-25 cell lines shared seven common proteins, which were selectively enriched in abundance under hypoxic exposure. These proteins are present at variable levels in HNSCC cell line-derived sEVs, with MLX and STON2 being the most abundant (Figure 2E). Interestingly, three of the seven proteins were not detected in HaCaT-derived sEVs regardless of the level of hypoxia (Figure 2D,E). Among the proteins detected in the HNSCC-derived sEVs, we identified the potent angiogenesis-inducing protein Dysferlin, endocytosis regulator Stonin 2, and a protein encoded by the KT33B gene with structural integrity activity. Proteins encoded by MLX, LIPA3, and NEK5 were detected both in hypoxic HNSCC- and HaCaT-derived sEVs and are involved in the cellular energy metabolism. The detected protein encoded by P12L1 gene was found to be undefined as yet (Figure 2F). Our results indicate that the discovered protein signature consisting of the seven proteins listed in Figure 2F could be potentially used to discriminate between tumor cells exposed to normoxic or hypoxic conditions, while three of them representing only HNSCC-derived sEVs could serve as carcinogenic markers associated with hypoxia.

### 3.3. The Degree of Hypoxia in Tumor Cells Is Reflected in the Protein Profile of HNSCC-Derived sEVs

**Figure 3:**
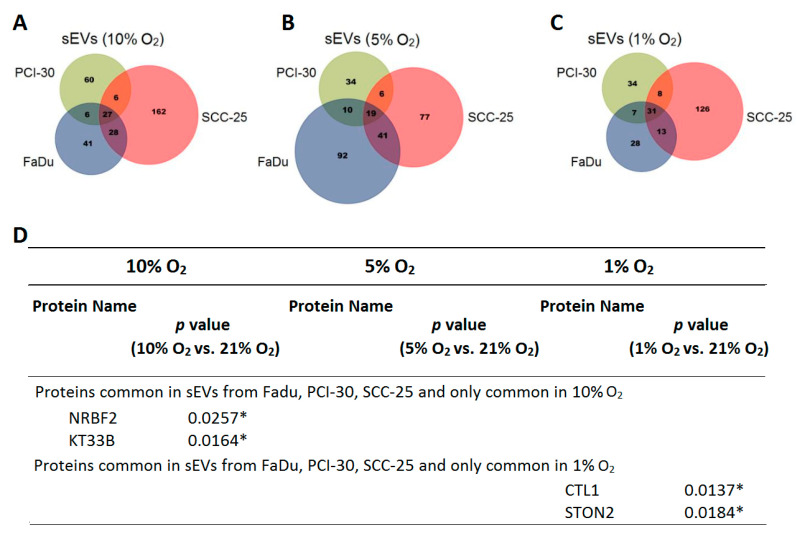
Venn diagrams show numbers of proteins detected in Head and Neck Squamous Cell Carcinoma (HNSCC)-derived small extracellular vesicles (sEVs) obtained from supernatants of the cells exposed to (**A**) 10% O_2_ (**B**), 5% O_2,_ and (**C**) 1% O_2_. (**D**) Protein profile in indicated degrees of hypoxia. Kruskal-Wallis test was used to compare the difference in intensities between oxygenation groups of their expression vs. normoxia. To isolate differences between groups Benjamini-Krieger-Yekutieli two-stage linear step-up procedure was performed. Differences were considered significant at * *p* < 0.05. Full table with detected proteins shared by hypoxic HNSCC-derived sEVs is presented in Table S2.

Since the above-mentioned signature panel only discriminates between normoxic or hypoxic conditions, but HNSCCs are characterized by heterogenous degrees of hypoxia within the tumor tissue, we further analyzed whether sEVs reflect the degree of hypoxia in tumor cells. Analysis of the protein cargo of HNSCC-derived sEVs revealed 27, 19, and 31 proteins shared by FaDu, PCI-30, and SCC-25, when exposed to 10, 5, and 1% O_2_, respectively (Figure 3A–C). Further analysis of these shared proteins revealed ten proteins, which were common between all cell lines independently of the degree of hypoxia, two proteins (FETUA, DLG5) that were commonly present after exposure to lower oxygen levels (1% and 5%) and two proteins (CLCF1, TBCD1) that were commonly present after exposure to higher oxygen levels (5% and 10%). Eight proteins were absent in moderate degrees of hypoxia (5%), but present in 1% and 10% of hypoxia (Table S2). Analysis of the individual groups revealed that the proteins KT33B and NRBF2 were significantly enriched in sEVs deriving from HNSCC cells cultured in 10% O_2_ compared to their normoxic counterparts (*p* = 0.0164 and *p* = 0.0257, respectively). In severe hypoxia (1% O_2_) HNSCC-derived sEVs were significantly enriched in STON2 (*p* = 0.0184) and CLT1 (*p* = 0.0137) compared to normoxic sEVs (Figure 3D). Thus, quantitative or semiquantitative analysis of protein profiles in HNSCC-derived sEVs could differentiate tumors with high hypoxia from tumors with low hypoxia, which would be of clinical relevance.

### 3.4. Functional Gene Designations for the Identified Proteins

**Figure 4:**
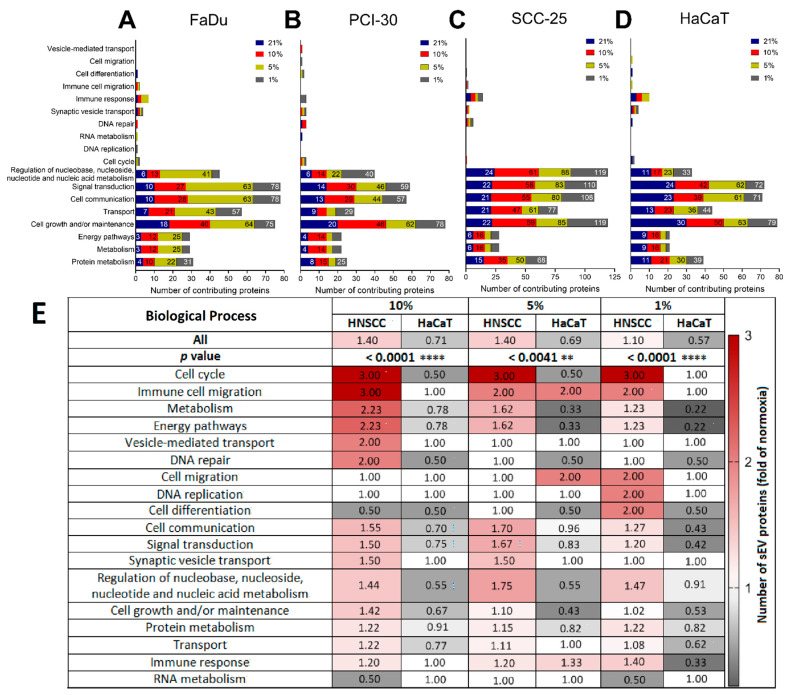
Proteome profile distribution of sEVs deriving from (**A**) FaDu, (**B**) PCI-30, (**C**) SCC-25, and (**D**) HaCaT cells cultured in various oxygen concentrations (21, 10, 5, and 1% O_2_) according to their major functional FunRich annotations. (**E**) Dataset of cancerous and non-cancerous sEV proteins presented as fold change of normoxia with regards to changes in contributions to all and specific biological processes, **** *p* < 0.0001, ** *p* < 0.01.

The identified proteins were analyzed by defining their participation in biological processes according to the FunRich analysis tool. sEVs derived from all evaluated cell lines mainly carried proteins that contribute to cell growth and maintenance, signal transduction and cell communication independently of the oxygenation status of the parent cells. Importantly, proteins belonging to biological processes such as energy pathways and metabolism or protein and nucleic acid regulation were enriched in sEVs (Figure 4A–D). To demonstrate the intensity of hypoxia-induced alterations the mean fold change of normoxia was calculated (Figure 4E). The abundance of proteins contributing to the above-mentioned pathways was increased in HNSCC cell line-derived sEVs when the parent cells were exposed to different levels of hypoxia compared to sEVs deriving from normoxic cultures (fold of normoxia: 1.4 for 10% O_2_; 1.4 for 5% O_2_; 1.1 for 1% O_2_; Figure 4E). In contrast, protein numbers were reduced in sEVs isolated from HaCaT cells exposed to hypoxia (fold of normoxia: 0.71 for 10% O_2_; 0.69 for 5% O_2_; 0.57 for 1% O_2_). sEVs isolated from hypoxic HNSCC cultures were mostly enriched in proteins that regulate the cell cycle, transport, metabolism, or energy pathways, whereas downregulation of these proteins was observed in HaCaT cells cultured in hypoxic conditions (*p* < 0.0001 vs. HaCaT) (Figure 4E). In all analyzed cell lines, only a small number of proteins were associated with immune-related functions (Figure 4A–D). However, the abundance of these proteins was increased in all hypoxic groups of HNSCC-derived sEVs, although not reaching statistical significance (*p* = 0.0842 vs. HaCaT), and increased in sEVs deriving from HaCaT cells cultured in 5% O_2_ (Figure 4E).

Analogous to the hypoxia-mediated changes in the protein profile of HNSCC-derived sEVs, we observed that the different levels of hypoxia resulted in specific functional patterns. sEVs isolated from HNSCC cells cultured in 10% O_2_ were enriched in proteins associated with vesicle-mediated transport and DNA repair (*p* = 0.0377 vs. HaCaT). sEVs from HNSCC cells cultured in 1% O_2_ were enriched in proteins regulating DNA replication, cell migration, and cell differentiation (*p* = 0.0022 vs. HaCaT; Figure 4E). These findings indicate that the degree of hypoxia on tumor cells leads not only to unique protein profiles as described above, but also to potential functional differences of sEVs.

### 3.5. sEVs Isolated from HNSCC Cells Cultured in Hypoxic Conditions Are Enriched in Angiogenic Proteins

**Figure 5:**
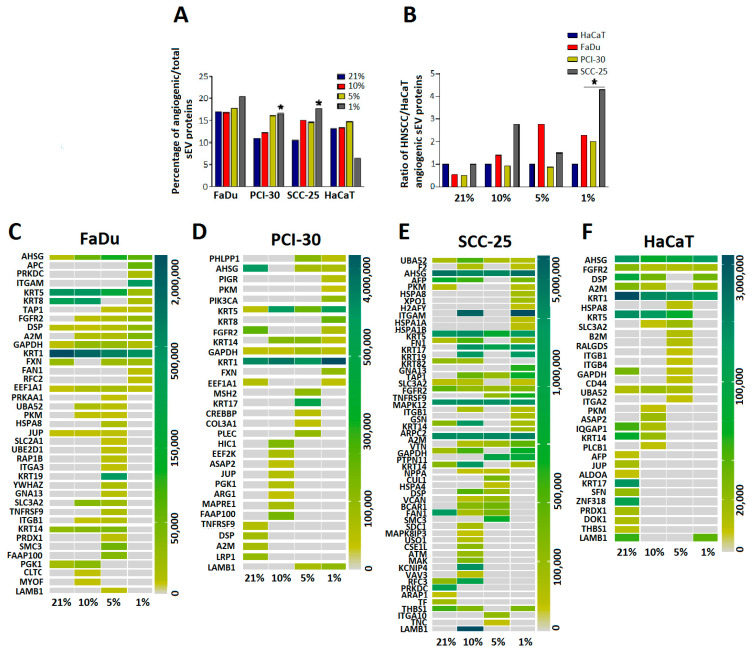
Characterization of pro-angiogenic proteins in small extracellular vesicles (sEVs). (**A**) Ratio of angiogenesis-related proteins to all proteins detected in sEVs deriving from indicated cell lines. * *p* < 0.05 vs. 21% O_2_. (**B**) Ratio of angiogenesis-related proteins in Head and Neck Squamous Cell Carcinoma (HNSCC)-derived sEVs to Human Keratinocyte (HaCaT)-derived sEVs, * *p* < 0.05 vs. HaCaT. (**C**–**F**) Abundance of angiogenesis-related proteins in sEVs deriving from indicated cell lines and oxygen levels.

Hypoxia is the main stimulus for the formation of tumor angiogenesis and, therefore, for the ingrowth of new blood vessels into the tumor. Since sEVs were previously shown to stimulate angiogenesis by transporting pro-angiogenic molecules to endothelial cells [17], we next asked whether angiogenesis-related proteins were present in sEVs and whether these proteins were modulated by oxygen levels. We found that sEVs from all cell lines were enriched in angiogenic proteins regardless of the culture conditions of the parent cells and represented approximately 10–15% of all detected proteins in sEVs (Figure 5A). Interestingly, the ratio of angiogenic proteins/all detected sEV proteins increased dose-dependently in sEVs derived from all three HNSCC cell lines when oxygen levels of the cell culture were lowered (Figure 5A). For HaCaT-derived sEVs, this increase was not observed and the culture in 1% O_2_ even decreased the proportion of angiogenic proteins to all sEV proteins (Figure 5A). Next, we compared the relation of angiogenic proteins between cancerous and non-cancerous cell line-derived sEVs isolated from cultures exposed to different degrees of hypoxia. sEVs isolated from FaDu and SCC-25 were enriched in angiogenic proteins compared to sEVs isolated from HaCaT cells exposed to 10 and 5% O_2_. In severe oxygen deficiency (1% O_2_) the amount of proangiogenic sEV proteins was significantly elevated in all HNSCC-derived sEVs when compared to HaCaT-derived sEVs (*p* < 0.05; Figure 5B).

To determine which pro-angiogenic proteins are packaged into sEVs in response to hypoxic conditions, the proteins known to participate in angiogenesis-related pathways were quantified and compared to sEVs that were generated in normoxic conditions. This analysis confirmed the increased numbers of angiogenesis-related proteins in sEVs isolated from all HNSCC cell lines cultured with 10, 5, or 1% O_2_. The detailed list of detected pro-angiogenic proteins and their abundance is presented in the heatmaps in Figure 5C–F. HNSCC cell line-derived sEVs showed a more variable composition of pro-angiogenic proteins compared to HaCaT-derived sEVs, especially in cultures exposed to 1% O_2_ (Figure 5C–F).

**Figure 6:**
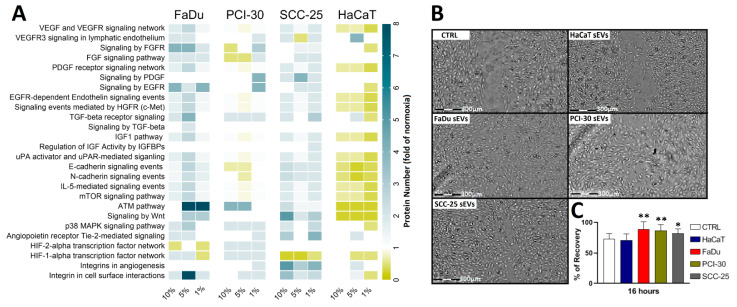
Hypoxic conditions stimulate the release of small extracellular vesicles (sEVs) from Head and Neck Squamous Cell Carcinoma (HNSCC) cells with pro-angiogenic functions. (**A**) Number of proteins related to pro-angiogenic pathways identified in sEVs produced by HNSCC cells exposed to various levels of hypoxia. Data are presented as fold of normoxia (21% O_2_). (**B**) Representative microscope images of wound healing assay of Human Umbilical Vein Endothelial Cells (HUVECs) co-incubated for 16 h with 3 μL phosphate buffered saline (CTRL) or 3 µg of sEVs at a concentration of 1 µg/µL isolated from 5% O_2_ HaCaT, FaDu, PCI-30, SCC-25. (**C**) Quantification of wound closure. All values represent means ± SEM (* *p* < 0.05; ** *p* < 0.01).

The upregulated proteins were related to several angiogenic pathways, predominantly the VEGF and VEGFR signaling network, HGFR signaling, EGFR-dependent Endothelin signaling, the ATM pathway, and Wnt signaling (Figure 6A). Despite the increase in number of proteins involved in regulation of angiogenesis, the differences in their intensities between oxygen levels did not reach significance. In contrast to the HNSCC cells, sEVs isolated from HaCaT cells cultured in hypoxic conditions showed downregulated abundance of most angiogenesis-related proteins, except integrins, angiopoietin receptor Tie-2-mediated signaling, VEGFR3 signaling in lymphatic endothelium, and TGF-β signaling-associated proteins, which were up-regulated in sEVs isolated from HaCat cells exposed to 5% O_2_ (Figure 6A).

Since sEVs isolated from HNSCC cells exposed to 5% O_2_ were mostly enriched in pro-angiogenic proteins, we evaluated their effects on endothelial cells (HUVECs) using the wound healing assay as an in vitro angiogenesis model. Co-incubation with 3 µg of sEVs stimulated HUVEC motility compared to CTRL. After 16 h of co-incubation, all HNSCC-derived sEVs induced wound closure (*p* < 0.05), whereas no difference was observed comparing HaCaT-derived sEVs with CTRL (Figure 6B,C). These findings demonstrate that the hypoxia-induced proteomic alterations in HNSCC-derived sEVs translate into biological functions.

## 4. Discussion

Tumor hypoxia is a hallmark in HNSCC and is considered to drive tumor progression by establishing a malignant phenotype, as well as promoting resistance to anti-cancer therapies [31]. Tumor cells exposed to hypoxic conditions are characterized by genomic/proteomic alterations, ultimately resulting in distinct functions. These cellular processes are reflected in tumor-derived sEVs, which are released more abundantly in hypoxic conditions and show distinct cargo components and functions [25,32,33]. To fully understand the hypoxic landscape in HNSCC, which is characterized by heterogenous levels of tissue hypoxia, novel diagnostic approaches are required to capture most detailed information about the tissue environment. In this regard, tumor-derived sEVs might be a promising resource for monitoring the hypoxic environment in HNSCC and potentially could predict response to anti-cancer therapies, such as anti-angiogenic or vascular normalization therapies [34].

To get a better understanding of the sEV cargo components in hypoxic conditions, we simulated the heterogenous levels of tissue hypoxia in HNSCC by gradually decreasing oxygen levels in our cell cultures. These studies provided the in vitro evidence that the release of sEVs by HNSCC cells is increased in response to hypoxia and that the proteomic profiles shift depending on the oxygenation status of the parent cells. We were able to identify novel protein signatures which are uniquely present in sEVs released in hypoxic conditions. So far, screening for hypoxic signatures in HNSCC revealed several tissue biomarkers, including HIF-1α, HIF-2α, carbonic anhydrase IX, GLUT1, and osteopontin [35]; however, these markers depend on the histological evaluation of tumor tissue and may only give information about the local oxygenation status, rather than capturing the heterogenous hypoxic landscape present in HNSCC. Alternatively, imaging by position emission tomography (PET) is being used to assess the heterogeneity of tumor hypoxia [36]. Analyzing the onco-proteome carried by sEVs additionally enables to get insights into the hypoxia-triggered molecular characteristics of the tumor cells and may even identify novel putative therapeutic targets [37]. Just looking at tumor-derived sEVs, unique protein signatures reflect the degree of hypoxia present in the cellular environment, even differentiating between low, moderate, and severe hypoxia. Thus, analyzing the complex molecular and genetic cargo components of sEVs may have wider possibilities than tumor hypoxia imaging itself. In a recent study, a HNSCC patient cohort was analyzed classifying patients with more or less hypoxic tumors based on hypoxic gene signatures [38]. Analogous to these findings, hypoxic gene signatures were also identified in sEVs and were shown to predict recurrence in lung adenocarcinoma [39].

To date, only a few studies of the sEV proteome as a source of novel biomarkers have been reported, although emerging evidence underlines the potential of using this resource in HNSCC. Screening for onco-proteomic sEV-based biomarkers in HNSCC provided the first evidence that immunoregulatory proteins such as PD-1, PD-L1, and CTLA-4 can be utilized as markers of tumor progression and might play an important functional role due to their immunosuppressive effects [40]. Since our study is cell line-based, the presented results need to be validated using tumor and sEV samples from HNSCC patients. According to the literature, some of the components in the hypoxic protein signatures we report are known to be involved in hypoxia-related events and show prognostic relevance in cancer. For instance, dysferlin was described to be a proteomic marker of muscle dystrophies [41] and introduced as a promising prognostic biomarker in clear cell renal cell carcinoma [42]. Stonin-2 overexpression was correlated with unfavorable prognosis and tumor invasion in epithelial ovarian cancer [43]. CTL1 was found to be strongly expressed in colon, breast, and lung carcinoma [44]. Investigating the clinical potential of these markers in HNSCC—in tumor tissue as well as in plasma-derived sEVs—will be an aim for our future studies.

## 5. Conclusions

The application of non-invasive liquid biopsies in HNSCC is of great current interest and several approaches have been considered in recent years, including the analysis of ctDNA, circulating tumor cells, and sEV-associated microRNAs [45]. However, the concept of using proteomic profiles of sEVs isolated from the plasma of cancer patients as diagnostic/prognostic biomarkers was recently validated in several human cancers [18,19]. Analogous, our findings indicate that cargo components of sEVs are a valuable resource for assessing the degree of hypoxia in cancerous tissues because (1) exposure of parent cells to different levels of hypoxia stimulates secretion levels of sEVs and alters their proteomic cargo, (2) tumor-derived sEVs released under hypoxic conditions carry unique proteomic signatures, which may serve as a valuable resource for assessing tissue oxygenation, (3) HNSCCs are characterized by heterogenous degrees of tissue hypoxia, which are reflected in the proteomic cargo composition of sEVs, and (4) hypoxia-derived sEVs are enriched in pro-angiogenic proteins. Future studies are necessary to directly correlate the abundance of the sEV-based protein signatures with tissue gene/protein expression patterns and, therefore, potentially translate our findings to clinical samples.

## Data Availability

Raw data of LC-MS/MS analysis is available at the link: https://wum-my.sharepoint.com/:f:/g/personal/alicja_gluszko_wum_onmicrosoft_com/EhMdBsd5-TpLpTWwKAWrED8BKDnNEnCpaLcimBwX_IyUvw?e=munWZb (accessed on 13 August 2021), with the password “13082021”.

## Supplementary Materials

The following are available online at https://www.mdpi.com/article/10.3390/cancers13164176/s1, Figure S1: Representative concentration and size distribution plot of HNSCC and HaCaT-derived sEVs measured by nanoparticle tracking analysis (NTA) and particle visualization based on Brownian motions, Figure S2: Whole blot image of CD63 western blot shown in Figure 1C, Figure S3: Whole blot image of CD9 western blot shown in Figure 1C, Figure S4: Whole blot image of Grp94 western blot shown in Figure 1C, Table S1: Overview of the selected HNSCC cell lines with regards to anatomical sites of origin, estimated incidence, clinical and molecular characteristics concerning response to anti-angiogenic therapies, Table S2. Profile of all proteins in indicated degrees of hypoxia.
