# Supplementary material for: Small Extracellular Vesicles from Head and Neck Squamous Cell Carcinoma Cells Carry a Proteomic Signature for Tumor Hypoxia
Source: Cancers (Basel). 2021 Aug 19;13(16):4176. doi: 10.3390/cancers13164176 (PMC8393921; doi:10.3390/cancers13164176)
Supplement: Supplementary file 1 [file cancers-13-04176-s001.zip › cancers-1345395-supplementary.pdf]

Supplementary Materials

Small Extracellular Vesicles from Head and Neck Squamous Cell Carcinoma Cells Carry a Proteomic Signature for Tumor Hypoxia

Alicja Głuszko, Mirosław J. Szczepański, Theresa L. Whiteside, Torsten E. Reichert, Jacek Siewiera and Nils Ludwig

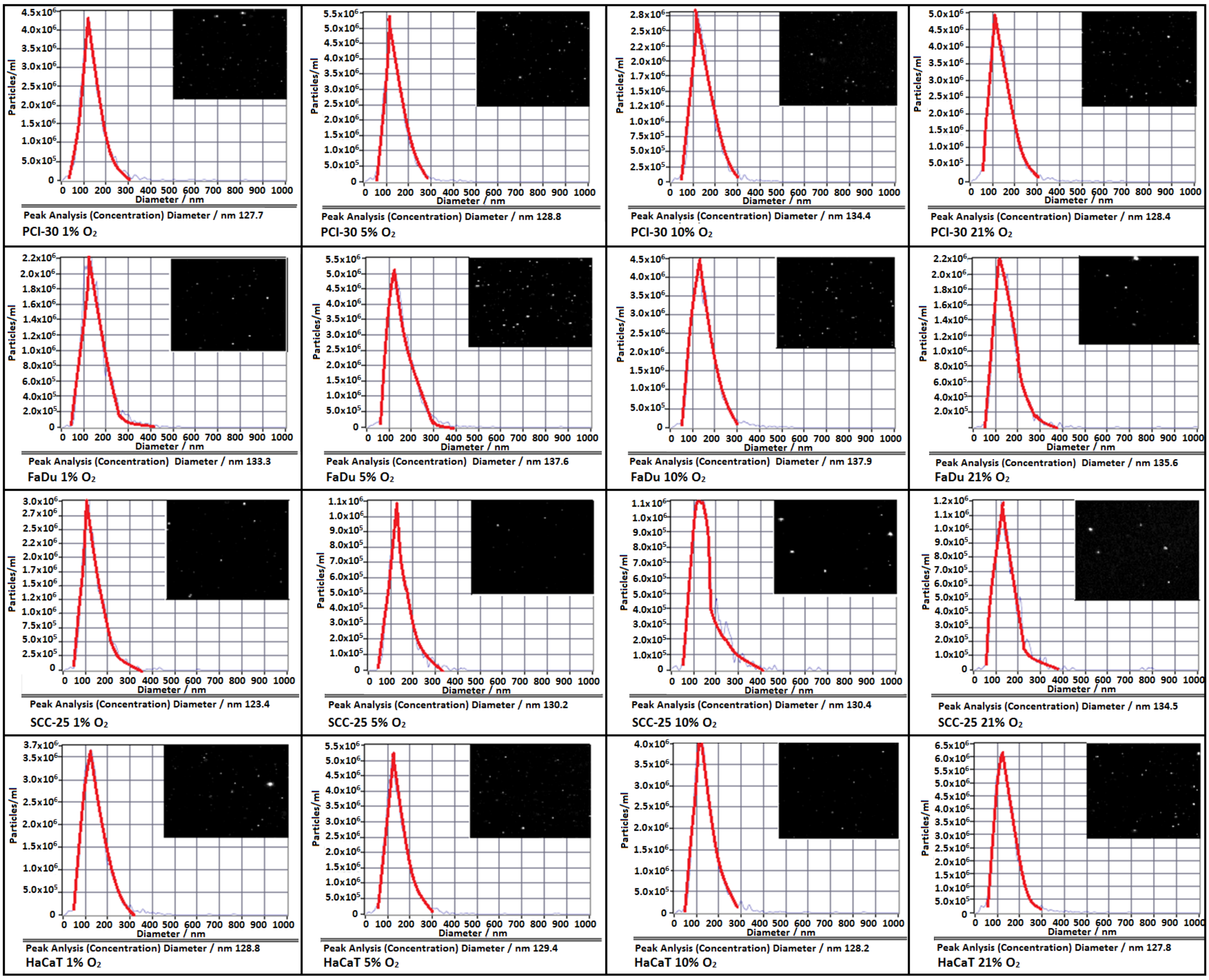

Figure S1. Representative concentration and size distribution plot of HNSCC and HaCaT-derived sEVs measured by nanoparticle tracking analysis (NTA) and particle visualization based on brownian motions. Particle concentration and diameter related to normoxic (21% O_2) and hypoxic (1% O_2, 5% O_2, 10% O_2) conditions.

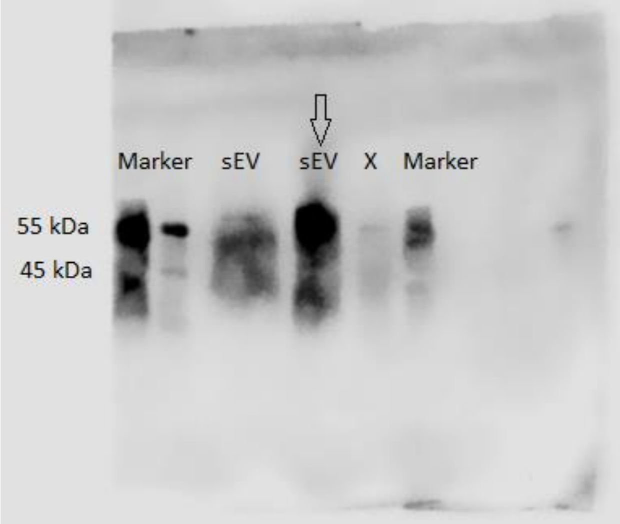

Figure S2. Whole blot image of CD63 western blot shown in Figure 1C. Arrow indicates the lane presented in Figure 1C.

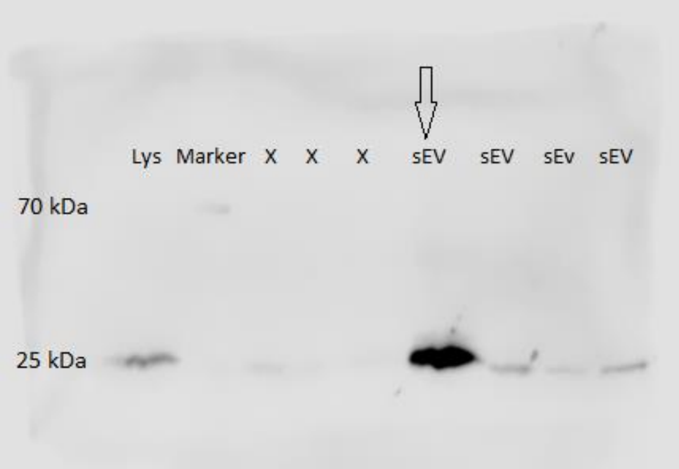

Figure S3. Whole blot image of CD9 western blot shown in Figure 1C. Arrow indicates the lane presented in Figure 1C.

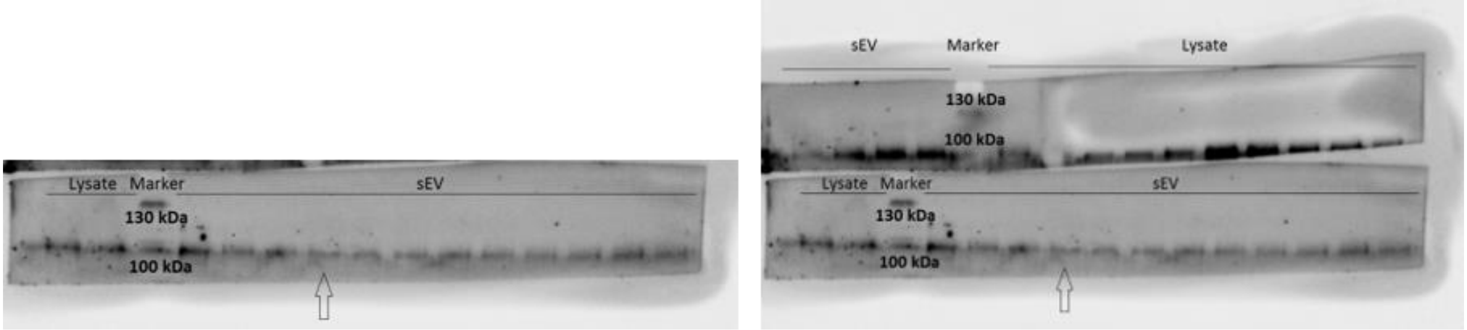

Figure S4. Whole blot image of Grp94 western blot shown in Figure 1C. Arrow indicates the lane presented in Figure 1C.

Table S1. Overview of the selected HNSCC cell lines with regards to anatomical sites of origin, estimated annual number of incidences, clinical and molecular characteristics concerning response to anti-angiogenic therapies.

HNSCC Cell Lines	Anatomical Site of Origin/Annual Number of Incidences	Aggressiveness/TNM stage/Characteristic	EGFR Expression/EGFR-Dependent Cell-Mediated Cytotoxicity	VEGFRs Expression/Response to VEGF Targeting Anti-Angiogenic Therapies	References
**FaDu**	Hypopharynx/84,254	High/-/Fast growing, highly invasive, forms large tumor	Differently high/high resistance	Positive/increased VEGF level	[1–5]
**PCI-30**	Oral cavity/377,713	High/T3N1M0/Fast growing, highly invasive	Differently high/susceptible	-/-	[1,5–7]
**SCC-25**	Oral cavity/377,713	Low/T2N1/Slowly growing, less invasive	Differently high/intermediate sensitivity	Positive, strong/-	[1,4,5,8]

TNM—Tumor size Nodus Metastases classification; T3N1M0—Tumor size 3, Nodus 1, Metastases 0 scale; T2N1—Tumor size 2, Nodus 1 scale; EGFR—Epidermal Growth Factor Receptor; VEGFR—Vascular Endothelial Growth Factor Receptor; VEGF—Vascular Endothelial Growth Factor.

Table S2. Profile of all proteins of small extracellular vesicles (sEVs) in indicated degrees of hypoxia. Kruskal-Wallis test was used to compare the difference in intensities between oxygenation groups of their expression vs. normoxia. To isolate differences between groups Benjamini-Krieger-Yekutieli two-stage linear step-up procedure was performed. Differences were considered significant at *$p < 0.05$.

**Hypoxia Signatures**					
**10% O_2**		**5% O_2**		**1% O_2**	
**Protein Name**	***p* value (10% O_2 vs. 21% O_2)**	**Protein Name**	***p* value (5% O_2 vs. 21% O_2)**	**Protein Name**	***p* value (1% O_2 vs. 21% O_2)**
Proteins common in sEVs from FaDu, PCI-30, and SCC-25 and common in all degrees of hypoxia					
STON1	0.4280	STON1	0.1742	STON1	0.7341
PCLO	0.3082	PCLO	0.3082	PCLO	>0.9099
CCD25	0.1742	CCD25	0.9099	CCD25	0.9099
K1C9	0.0894	K1C9	0.4969	K1C9	0.5713
K2C5	0.4969	K2C5	0.4280	K2C5	0.9099
K1C10	0.7341	K1C10	0.2575	K1C10	0.3082
ACTBL	0.6506	ACTBL	0.4280	ACTBL	0.4280
CCD87	0.9099	CCD87	0.9099	CCD87	0.8208
ALBU	0.9099	ALBU	0.6506	ALBU	0.7341
K2C1	>0.9999	K2C1	0.2129	K2C1	0.7341
Proteins common in sEVs from FaDu, PCI-30, and SCC-25 and common in 10% and 5% O_2					
CLCF1	0.1712	CLCF1	0.1382	-	-
TBCD1	0.4248	TBCD1	0.6483	-	-
Proteins common in sEVs from FaDu, PCI-30, and SCC-25 and common in 5% and 1% O_2					
-	-	FETUA	0.8208	FETUA	>0.9999
-	-	DLG5	0.6506	DLG5	0.1742
Proteins common in sEVs from FaDu, PCI-30, and SCC-25 and common in 10% and 1% O_2					
K22E	0.4969			K22E	0.7341
SNX29	0.4280			SNX29	0.8208
AHNK	>0.9999			AHNK	0.6506
ACTB	0.1382			ACTB	0.1104
S2542	0.9097			S2542	>0.9999
HBB	0.4802			HBB	0.6827
G3P	0.5713			G3P	0.5713
ABHD8	0.4610			ABHD8	0.6914
Proteins common in sEVs from FaDu, PCI-30, and SCC-25 and only common in 10% O_2					
ANO1	0.8627				
CE350	0.0603				
NRBF2	0.0257 *				
NSUN4	0.1340	-	-	-	-
K1C14	0.1382				
KT33B	0.0164 *				
HORN	0.1383				
Proteins common in sEVs from FaDu, PCI-30, and SCC-25 and only common in 5% O_2					
		TRI18	0.8196		
		TRY3	0.8205		
-	-	ZN714	0.3043	-	-
		VAT1	0.9097		
		AL1B1	0.0838		
Proteins common in sEVs from FaDu, PCI-30, and SCC-25 and only common in 1% O_2					
				TRFL	0.7732
				HBA	0.9092
				PI3R4	0.8196
				CTL1	0.0137 *
				K2C8	0.0698
-	-	-	-	TTLL1	0.9038
				FGFR2	0.8196
				DKK2	>0.9999
				CCD73	0.8196
				STON2	0.0184 *
				SHRM3	0.5261

sEVs—small extracellular vesicles; FaDu, PCI-30, SCC-25—Head and Neck Squamous Cell Carcinoma cell lines.

References

1. Lin, C.J.; Grandis, J.R.; Carey, T.E.; Gollin, S.M.; Whiteside, T.L.; Koch, W.M.; Ferris, R.L.; Lai, S.Y. Head and neck squamous cell carcinoma cell lines: established models and rationale for selection. *Head Neck*. **2007**; *29*, 63–88.
2. Patel, N.; Able, S.; Allen, D.; Fokas, E.; Cornelissen, B.; Gleeson, F.V.; Harris, A.L.; Vallis, K.A. Monitoring response to anti-angiogenic mTOR inhibitor therapy in vivo using (111)In-bevacizumab. *EJNMMI Res*. **2017**; *7*, 49.
3. Kalinowski, F.C.; Giles, K.M.; Candy, P.A.; Ali A.; Ganda C.; Epis M.R.; Webster, R.J.; Leedman, P.J. Regulation of epidermal growth factor receptor signaling and erlotinib sensitivity in head and neck cancer cells by miR-7. *PLoS One*. **2012**; *7*, e47067.
4. Lalla, R.V.; Boisoneau, D.S.; Spiro, J.D.; Kreutzer, D.L. Expression of vascular endothelial growth factor receptors on tumor cells in head and neck squamous cell carcinoma. *Arch Otolaryngol Head Neck Surg*. **2003**; *129*, 882–888.
5. GLOBOCAN. Cancer Today: Estimated number of incident cases worldwide, both sexes, all ages. Available online: https://gco.iarc.fr/today/online-analysis-multi-bars?v=2020&mode=cancer&mode_population=countries&population=900&populations=900&key=total&sex=0&cancer=39&type=0&statistic=5&prevalence=0&population_group=0&ages_group%5B%5D=0&ages_group%5B%5D=17&nb_items=55&group_cancer=1&include_nmsc=1&include_nmsc_other=1&type_multiple=%257B%2522inc%2522%253Atrue%252C%2522mort%2522%253Afalse%252C%2522prev%2522%253Afalse%257D&orientation=horizontal&type_sort=0&type_nb_items=%257B%2522top%2522%253Atrue%252C%2522bottom%2522%253Afalse%257D (accessed on 13 Aug 2021).
6. Szczepanski, M.J.; Czystowska, M.; Szajnik, M.; Harasymczuk M.; Boyiadzis, M.; Kruk-Zagajewska A.; Szyfter, W.; Zeromski, J.; Whiteside, T.L. Triggering of Toll-like receptor 4 expressed on human head and neck squamous cell carcinoma promotes tumor development and protects the tumor from immune attack. *Cancer Res.* **2009**; *69*, 3105–3113.
7. Lopez-Albaitero A, Lee SC, Morgan S, Grandis JR, Gooding WE, Ferrone S, Ferris, R.L. Role of polymorphic Fc gamma receptor IIIa and EGFR expression level in cetuximab mediated, NK cell dependent in vitro cytotoxicity of head and neck squamous cell carcinoma cells. Cancer Immunol Immunother. **2009**; *58*, 1853–1864.
8. Dourado, M.R.; Korvala, J.; Astrom, P.; De Oliveira, C.E.; Cervigne, N.K.; Mofatto, L.S.; Campanella Bastos, D.; Pereira Messetti, A.C.; Graner, E.; Paes Leme, A.F. et al. Extracellular vesicles derived from cancer-associated fibroblasts induce the migration and invasion of oral squamous cell carcinoma. *J Extracell Vesicles*. **2019**; *8*, 1578525.

© 2021 by the authors. Licensee MDPI, Basel, Switzerland. This article is an open access article distributed under the terms and conditions of the Creative Commons Attribution (CC BY) license (http://creativecommons.org/licenses/by/4.0/).
